# Secreted Protein Acidic and Rich in Cysteine as A Regeneration Factor: Beyond the Tissue Repair

**DOI:** 10.3390/life11010038

**Published:** 2021-01-08

**Authors:** Abdelaziz Ghanemi, Mayumi Yoshioka, Jonny St-Amand

**Affiliations:** 1 Department of Molecular Medicine, Faculty of Medicine, Laval University, Québec, QC G1V 0A6, Canada; abdelaziz.ghanemi@crchudequebec.ulaval.ca; 2 Functional Genomics Laboratory, Endocrinology and Nephrology Axis, CHU de Québec-Université Laval Research Center, Québec, QC G1V 4G2, Canada; mayumi.yoshioka@crchudequebec.ulaval.ca

**Keywords:** secreted protein acidic and rich in cysteine, regeneration, homeostasis

## Abstract

Diverse pathologies (inflammation, tissues injuries, cancer, etc.) and physiological conditions (obesity, physical activity, etc.) induce the expression/secretion of the matricellular protein, secrete protein acidic and rich in cysteine (SPARC). SPARC contributes to the creation of an environment that is suitable for tissue regeneration through a variety of roles, including metabolic homeostasis, inflammation reduction, extracellular matrix remodeling and collagen maturation. Such a homeostatic environment optimizes tissue regeneration and improves tissues’ repair ability. These properties that SPARC has within the regeneration contexts could have a variety of applications, such as in obesity, cancer, sarcopenia, diabetes and bioengineering.

Tissue regeneration is a vital process allowing organisms to overcome biological disturbances and adapt to changes and physiological development via the renewal, growth and restoration of diverse cells and tissues. The regeneration ability changes throughout the lifespan, which leads to diverse tissue malfunctions and diseases [1]. The regenerative process could be either normal or limited (abnormal) depending on the biological environment. Indeed, under healthy environmental conditions (stem cells growth ratio [2], growth factors [3], hormones [4,5], pH [6,7], etc.), the regenerative processes are optimized. They allow for regular tissue development and adaptation to the corresponding biological functions. However, under physiological (ageing [8,9]) or pathological (cancer [10], obesity [11], inflammation [12], etc.) conditions, or when impacted by disturbing stimuli or exogenous factors (such as radiations [13]), tissues’ regeneration ability and functions could be impaired. To overcome this “negative” regeneration environment, the organism has a variety of tools to compensate or reduce the intensity or the impacts. These correcting or counteracting mechanisms are mediated through what could be considered regeneration factors. Among these molecules, secreted protein acidic and rich in cysteine (SPARC) has a variety of roles and implications. One of the SPARC properties is its ability to optimize the regeneration environment with an improved cellular regenerative capacity from different perspectives (metabolics, tissue repair, oxidation, inflammation, cancer, etc.), as illustrated below.

SPARC, also known as BM-40 or osteonectin (32 kDa [14]), is a matricellular (extracellular matrix-associated) protein. Unlike its name (osteonectin) might suggest, SPARC expression is not limited to bones, but this glycoprotein is also present in diverse tissues including nonmineralized tissues, in platelets [15] and in muscles [16]. Such wide distribution correlates with SPARC roles during embryogenesis [17] as well as during tissue repair, cell turnover, cellular differentiation and remodeling [18,19,20,21,22], which are key steps in tissue regeneration. Therefore, SPARC expression or levels increase following injuries such as myocardial injury [23], myopathies [24] and in situations (either physiological or pathological) where tissues undergo changes (repair, renewal and remodeling) such as during obesity [18,25], skeletal muscle regeneration [26], cancer [27], systemic sclerosis, hepatic fibrosis [28] and physical exercise. Indeed, SPARC/*Sparc* expression increases in the skeletal muscle during training [29], as well as following electrical pulse stimulation in muscle cells (considered to be the in vitro equivalent of exercise) [30]. Such situations do represent a disturbance of the homeostasis that leads to a “negative” regeneration environment. Therefore, biological processes that overcome such a homeostatic disturbance, restore a suitable environment for regeneration and rescue the affected tissues to allow better developmental patters are required. Interestingly, the situations in which SPARC is overexpressed are mainly those requiring regeneration, either to repair tissues (injury) or adapt to tissue changes (obesity, exercised muscle, etc.). These specific patterns highlight SPARC as a regenerative factor. In addition, the importance of the extracellular matrix in regeneration suggests close interactions between SPARC, the extracellular matrix [31] and matricellular protein components such as thrombospondin-2 [32] during the regeneration process.

Tissue regeneration is a process that requires the implication of numerous cellular organelles and the use of energy. Thus, regeneration has metabolic and biochemical needs to which the cellular machinery has to adapt [33]. In this context, SPARC has been shown to be implicated in a variety of metabolic functions, such as glucose tolerance improvement [34], while it is also required for both glucose homeostasis maintenance and insulin secretion [35]. In the skeletal muscle, SPARC also seems to act towards improved metabolic properties and functions [18,24], including mitochondrial functions [30,36,37], which is of interest knowing the importance of the mitochondria during regeneration [38,39]. Importantly, our latest study suggests that exercise-induced muscle phenotype changes are SPARC-dependent [40]. These SPARC properties are also completed by their important roles in energy balance and storage. For instance, SPARC inhibits adipogenesis [41] and its inactivation leads to an enhancement of high-fat diet-induced obesity [42]. These patterns correlate with the role of SPARC in brown adipocyte activation and lipid usage in white adipocytes [43]. Such energy metabolism effects—in addition to optimizing the regeneration (synchronization)—also lead to increased energy usage, thus reducing the risk of obesity through increased energy expenditure. This represents another illustration of how SPARC counteracts the “negative” regeneration environment, since obesity itself represents a status of impaired regeneration [44]. Indeed, during obesity, many factors lead to such a “negative” regeneration environment due to all the conditions induced by or associated with obesity, such as inflammation, insulin resistance, metabolic disorders [45,46] and even stem cell changes [47,48], that impact regeneration. SPARC is extremely important for bone formation, remodeling and regeneration [14,32,49,50,51]. This is important as well, not only for the structural homeostasis, but also for both locomotion and, most importantly, the energy metabolism. Indeed, the skeletal muscle that governs most of the energy expenditure [52] is supported by the skeleton with which it forms the locomotor (musculoskeletal) system. Therefore, the good metabolic and contractile function (strength) of muscles would require homeostatic skeleton development due to the close ties between both bones and skeletal muscles, including synchronized development [53].

Furthermore, in addition to such metabolic implications, SPARC is also involved in other growth and homeostasis-related patterns, including cancer homeostasis. SPARC is overexpressed during cancer [27] and has been reported to have anti-cancer properties [54,55]. SPARC has also been shown to have interesting roles within the inflammatory processes [56,57]. It has anti-inflammatory properties [56] and can, for instance, protect from adverse cardiac inflammation during viral myocarditis [58]. These properties of controlling cancer and inflammation development would impact the microenvironment, contributing to an improved homeostasis. Moreover, SPARC is required for the immune system functions [59], which is relevant, for instance, during immune-modulatory therapy to support the regeneration of injured muscles [60] and muscle healing [61]. Importantly, more roles are yet to be explored in terms of SPARC contribution at the physiological levels, such as in cardiomyocyte contraction [23]. This cardiac role would improve the blood circulation for diverse cells, which are vital for tissue regeneration). In addition, the therapeutic practice of cardiac regeneration [2,62] could benefit from SPARC properties in cardiac regeneration [19,63] as well. Beyond the cardiac properties, SPARC has roles in the cardiovascular properties, as suggested by its production by both bone-marrow-derived cells during myocardial fibrosis (in left ventricular pressure overload) [64] and pericytes, with a possible role in postinfarct healing [65], which is supported by the possible classification of SPARC as a marker for vascular complications in pre-diabetics [66].

All these highlighted properties point to SPARC as a regeneration factor. It not only has significant roles in tissue repair or development but contributes directly and indirectly to generating a “positive” biological environment that optimizes regeneration, as summarized in the graphical abstract. Moreover, other factors that work towards reducing the regeneration ability, such as ageing [1,67] and oxidative stress [68,69], are also counteracted—at least indirectly—by SPARC effects. For instance, SPARC-induced increased muscle functions (including via interactions with actin in skeletal muscle [24]) and metabolism would increase the antioxidant effect induced by exercise [70]. This contributes to the improvement in the regeneration environment by decreasing the oxidative stress. Furthermore, an improved muscular function (including during exercise) would lead to reducing the accumulation of the lactic acid and, therefore, better control of the pH, which both impacts muscle fatigue [71,72] and represents another important factor for different cellular functions [73], including those related to regeneration [74,75]. In addition, ageing-induced collagen loss [76] would be counteracted via the roles of SPARC in collagen properties [77,78,79,80]. Moreover, many SPARC effects counteract ageing impacts. In this context, ageing is a factor that decreases the regeneration ability [67], and with which we see an increased risk of obesity [81], sarcopenia [82,83], osteoporosis [84], etc. This points to SPARC not only as a regeneration factor that counteracts the ageing-related decrease in regeneration ability, but also as a factor with key roles against ageing-induced conditions that lead to health problems including sarcopenia, obesity (a health problem that could increase with the ongoing COVID-19 crisis [85]) and osteoporosis, through metabolic, structural and functional roles, and the impacts SPARC has on the corresponding tissues and organs (muscles, adipose tissue, bone, etc.). Therefore, SPARC remains worth exploring in the ageing process and geriatric research. These examples represent additional illustrations of SPARC’s contribution to creating the optimal environment for regeneration, and further point to it as a regenerative factor.

These patterns show complimentary roles in terms of the implications of SPARC in tissue repair, and the diverse metabolic and homeostatic effects it mediates [86]. Importantly, the fact that SPARC is overexpressed during pathological situations such as obesity and cancer, as well as during physical activity (physiological adaptation), further indicates that it could represent feedback. Rather than a damaging factor, SPARC would aim to counteract/correct the negative impacts induced by the pathological situations such as inflammation and tissue damage through properties including regeneration ability, as illustrated for the skeletal muscle [87]. Indeed, conditions (pathological and physiological) that lead to impaired regeneration by creating a negative environment are the same conditions under which SPARC overexpression has been reported. Such overexpressed SPARC improves the regeneration ability and reduces the negative environment by inducing functional and metabolic enhancement at different tissues. These actions reverse, correct or reduce the impacts those initial conditions had on regeneration, which will lead to a SPARC-induced corrected regeneration ability.

This paper presents SPARC as a promising therapeutic tool in a variety of health conditions, ranging from metabolism and inflammation to obesity and sarcopenia. Importantly, SPARC could also be an option in the area of tissue engineering based on its involvement in and impacts on the regenerative processes, especially with the known implications of SPARC in the functions of stem cells [88,89], as well as other types of cells such as erythroid progenitors [90]. Thus, SPARC-related pathways also represent a potential pharmacological target to optimize therapies in regenerative medicine as an adjuvant to optimize the regeneration environment of the targeted tissues and organs.

## Data Availability

Not applicable.
